# Further evidence for microtubule-independent dimerization of TPPP/p25

**DOI:** 10.1038/srep40594

**Published:** 2017-01-11

**Authors:** J. Oláh, T. Szénási, S. Szunyogh, A. Szabó, A. Lehotzky, J. Ovádi

**Affiliations:** 1 Institute of Enzymology, Research Centre for Natural Sciences, Hungarian Academy of Sciences, Budapest, Hungary

## Abstract

Tubulin Polymerization Promoting Protein (TPPP/p25) is a brain-specific disordered protein that modulates the dynamics and stability of the microtubule network by its assembly promoting, cross-linking and acetylation enhancing activities. In normal brain it is expressed primarily in differentiated oligodendrocytes; however, at pathological conditions it is enriched in inclusions of both neurons and oligodendrocytes characteristic for Parkinson’s disease and multiple system atrophy, respectively. The objective of this paper is to highlight a critical point of a recently published Skoufias’s paper in which the crucial role of the microtubules in TPPP/p25 dimerization leading to microtubule bundling was suggested. However, our previous and present data provide evidence for the microtubule-independent dimerization of TPPP/p25 and its stabilization by disulphide bridges. In addition, our bimolecular fluorescence complementation experiments revealed the dimerization ability of both the full length and the terminal-free (CORE) TPPP/p25 forms, however, while TPPP/p25 aligned along the bundled microtubule network, the associated CORE segments distributed mostly homogeneously within the cytosol. Now, we identified a molecular model from the possible ones suggested in the Skoufias’s paper that could be responsible for stabilization of the microtubule network in the course of the oligodendrocyte differentiation, consequently in the constitution of the myelin sheath.

Recently in *Scientific Reports*, Skoufias and co-workers published a paper entitled *“Self protein-protein interactions are involved in TPPP*/*p25 mediated microtubule bundling”* in which a number of data are presented on the interaction of Tubulin Polymerization Promoting Protein (TPPP/p25) forms with tubulin and microtubule (MT)^1^. In our previous studies following the discovery of TPPP/p25 as a microtubule associated protein (MAP) our data support a model for TPPP/p25-induced MT bundling^2^,^3^,^4^,^5^. Our reported findings in relation to the structure and function of TPPP/p25 - relevant to the Skoufias’s paper^1^ - are as follows: i) it is a disordered protein with unstructured N- and C-termini straddling a flexible CORE region as detected by multinuclear magnetic resonance spectroscopy^6^; ii) its dimers display higher tubulin assembly activity than the monomers^4^; ii) it induces MT assembly coupled with bundling activity resulting in resistance against anti-MT agents^7^. These findings indirectly support a mechanism for the stabilization of the MT network by dimeric TPPP/p25.

The objective of this Comment paper is to highlight a critical point leading the authors to the suggestion as follows: “*the central folded domain of TPPP/p25 **following binding to microtubules** can drive homotypic protein-protein interactions leading to bundled microtubules”* (cf. Abstract)^1^. In fact, this hypothesis is based upon the observation that: *“Our biophysical analysis of all the fragments though did not support the formation of TPPP/p25 dimers in solution” (page 11).* Our biochemical, biophysical and immunological Please change analysis to analyses. have provided evidence for concentration-dependent dimerization of TPPP/p25 in the absence of MTs^4^, however, it is promoted by the presence of ligands such as GTP^4^ and the bivalent zinc ion^8^. We have also established the role of intermolecular disulphide bridges in the stabilization of the dimers^4^, and that in the presence of dithioerythritol (DTE) (its epimer, DTT was used in the commented paper) the detection of the dimer forms is ambiguous^4^,^8^. In the experiments presented in Skoufias’s paper^1^, DTT (1 mM) is present in the TPPP/p25 solutions including the stored stock solutions and the solutions used for the binding and polymerization assays. This condition favours neither the detection of the dimeric form nor the dimer-enhanced tubulin polymerization activity resulting in low polymerization potency (cf. Fig. 1C of ref. 1 versus Fig. 3B of ref. 5). In order to enhance the relative amount of the disulphide-stabilized dimers, in selected experiments the stock solutions of TPPP/p25 (200 μM) were pre-incubated at room temperature, and the dimers could be detected even by sodium dodecyl sulphate polyacrylamide gel electrophoresis (SDS-PAGE) (cf. Fig. 1) as demonstrated earlier^4^.

In addition, we detected the dimeric form by other approaches as well such as sandwich enzyme-linked immunosorbent assay (ELISA)^8^, size exclusion gel chromatography^4^ and isothermal titration calorimetry^4^. Now we have performed sandwich ELISA experiment with the N- and/or C-terminal free forms as earlier with the full length protein to establish the role of the unstructured terminal segments in the dimerization of the TPPP/p25. To validate the *in vitro* data obtained with isolated protein variants, we have performed *in vivo* experiments as well using bimolecular fluorescence complementation assay (mVenus BiFC), similarly as described in the commented paper^1^.

## Results

### Effect of truncation of TPPP/p25 on its dimerization

Our sandwich ELISA assay using mouse monoclonal TPPP/p25 antibody^9^ (mAb) labelled with and without biotin exclusively detects dimeric/oligomeric forms of TPPP/p25 in solution independently whether they are stabilized by disulphide bridges or not^8^.

**Figure 1:**
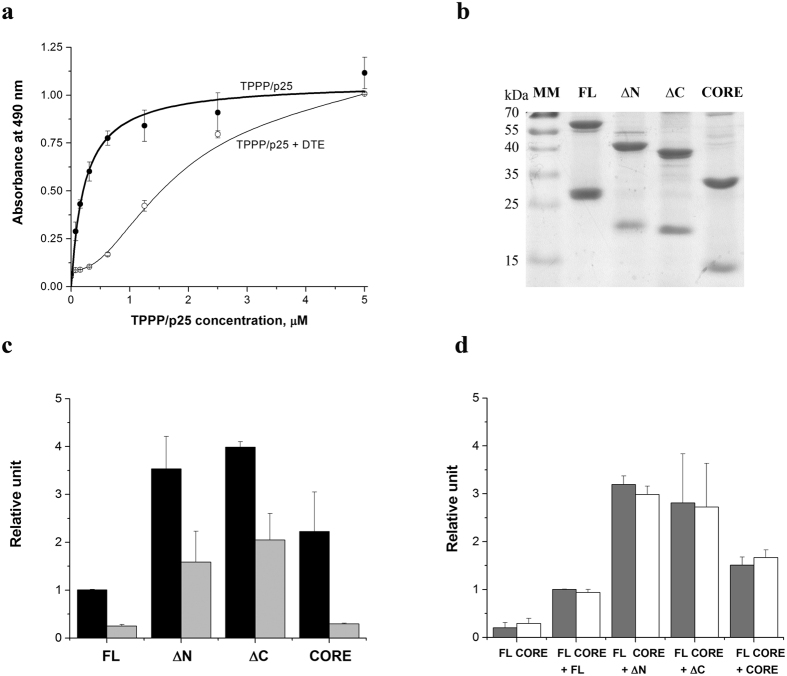
Effect of the truncation of TPPP/p25 on its dimerization. (**a**) Representative sandwich ELISA experiment with TPPP/p25 as presented previously^8^. Briefly, TPPP/p25 at different concentrations without or with preincubation with DTE was added onto the plate coated with mAb^9^; then the plate was sequentially incubated with biotinylated mAb and peroxidase conjugated avidin. (**b**) A representative image illustrating the formation of disulphide-stabilized dimers as detected by SDS-PAGE in the absence of DTE. MM: molecular weight marker. 5 μg protein from the stock solution (200 μM) were loaded into the gel in the absence of DTE. (**c**) Relative amount of the dimers obtained by sandwich ELISA (cf. Fig. 1a) with (light grey column) or without (black column) DTE. Different TPPP forms at 1 μM concentration were added to the plate coated with mAb in the absence and presence of DTE. (**d**) Dimerization of the different TPPP/p25 forms with full length (FL, grey column) or double truncated (CORE, white column) TPPP/p25 tested by sandwich ELISA. 1 μM FL TPPP/p25 or CORE TPPP/p25 was preincubated with 100 μM DTE; then the different TPPP/p25 forms were added, and dimers were detected by biotinylated mAb. (**a–d**) FL full length TPPP/p25, ΔN N-truncated TPPP/p25, ΔC C-truncated TPPP/p25, CORE double truncated TPPP/p25. Each data point represents the mean ± SD from three independent experiments (**a,c,d**).

Figure 1a shows a typical dissociation curve of the full length (FL) TPPP/p25 in the absence and presence of the reducing agent DTE. The finding is that the amount of the dimeric forms without reducing agent is much higher than with added DTE. Obviously, a fraction of the dimers stabilized by disulphide bridges in the stock solution does not dissociate into monomers.

To obtain information about the ratio of the stabilized dimers and monomers, aliquots from the stock solutions (200 μM) were loaded directly into SDS-PAGE. As shown in Fig. 1b, substantial fraction of all TPPP/p25 solutions contains disulphide-stabilized dimers. The stock solutions of the FL and the different truncated forms diluted to 1 μM were applied for sandwich ELISA experiments according to the following experimental design as illustrated schematically, the result are presented in Fig. 1c,d:


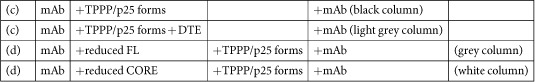


In one set of sandwich ELISA experiments the FL TPPP/p25 and its truncated forms were added to the immobilized mAb, and the dimer forms were quantified by biotinylated mAb (Fig. 1c). In the second set of experiments reduced FL or CORE form were added to the immobilized mAb, then the associations of the different TPPP/p25 forms with the monomeric FL or CORE forms were tested by the biotinylated mAb (Fig. 1d).

The sandwich ELISA data presented in Fig. 1c,d demonstrate the dimerization/oligomerization potency of all TPPP/p25 forms including the double truncated (CORE) one; in addition, the capability of both the reduced (monomer) FL and CORE forms to interact with the various TPPP/p25 forms, even if to different extents, is validated. This issue occurred in the absence of tubulin/MTs.

### Effect of TPPP/p25 truncation on the interaction with tubulin and MT

Now turbidity and pelleting measurements were performed with tubulin or paclitaxel-stabilized MTs using FL and N- and/or C-terminal truncated forms of TPPP/p25. In fact, similar types of experiments were carried out previously with tubulin^5^.

**Figure 2:**
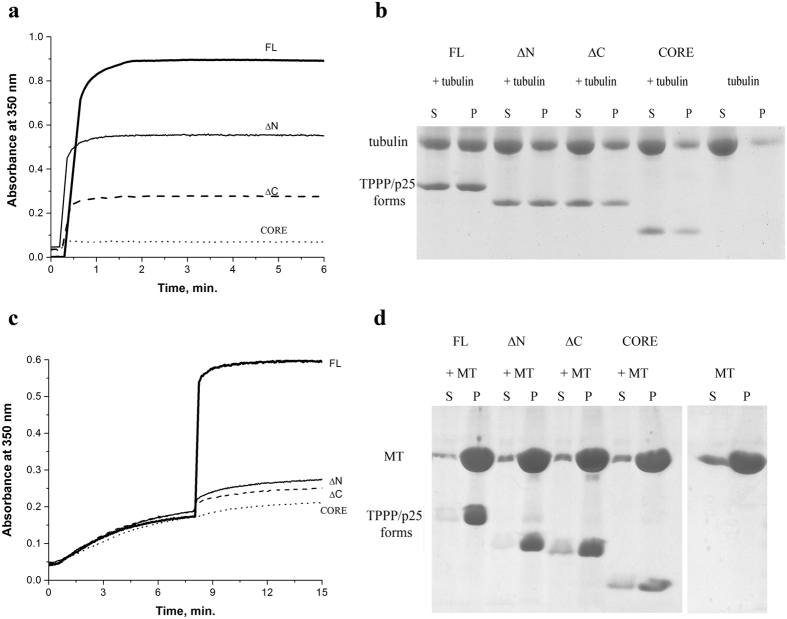
Tubulin polymerization and bundling potency of the TPPP/p25 forms as detected by turbidimetry (a,c) as well as by SDS/PAGE (b,d). (**a**) Tubulin assembly induced by the TPPP/p25 forms (turbidimetry); (**b**) pelleting of the assembled forms detected by SDS/PAGE in the presence of DTE; (**c**) cross-linking of paclitaxel-stabilized MT by TPPP/p25 forms; (**d**) co-pelleting of the TPPP/p25 forms with paclitaxel-stabilized MTs. Standard deviation (SD) was ± 10% (n = 3).

As shown in Fig. 2, the polymerization potency of the FL TPPP/p25 to both tubulin and MT is affected by the nature of the truncation. According to the Skoufias’s paper the order of the binding constants are published for the interaction of the MT with FL, N- or C-terminal free forms (cf. Fig. 4 in ref. 1)^1^. However, it needs to emphasise that there is a difference between the length of the CORE segments 50–157 *vs* 44–174, used in the two labs, consequently the lengths of the termini are shorter in our case. Nevertheless, the data presented in Fig. 2 qualitatively do not differ from that presented in the commented paper. According to our studies, the following qualitative order could be established for the binding and polymerization potencies of the TPPP/p25 forms:


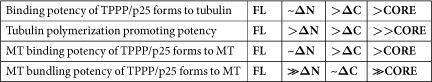


### Intracellular dimerization of TPPP/p25 and its CORE segments

As we revealed earlier TPPP/p25 co-localizes with the MT network and displays extensive bundling activity in different cell lines expressed by transfection (HeLa) or doxycycline induction (CHO10) or endogenously (CG-4), and it regulates the dynamics and organization of the MT network via its tubulin polymerization promoting and MT bundling activity as well as tubulin acetylation enhancing activity^5^,^7^,^10^. We have also shown with immunofluorescence microscopy that in contrast to the FL TPPP/p25 the CORE segment (double truncated form) distributed homogeneously within the cytoplasm^5^. Most importantly, we suggested that *“The finding that the double truncated TPPP/p25 cannot associate with the MT network underlines the role of the unstructured termini in the physiological function of TPPP/p25” *5.

**Figure 3:**
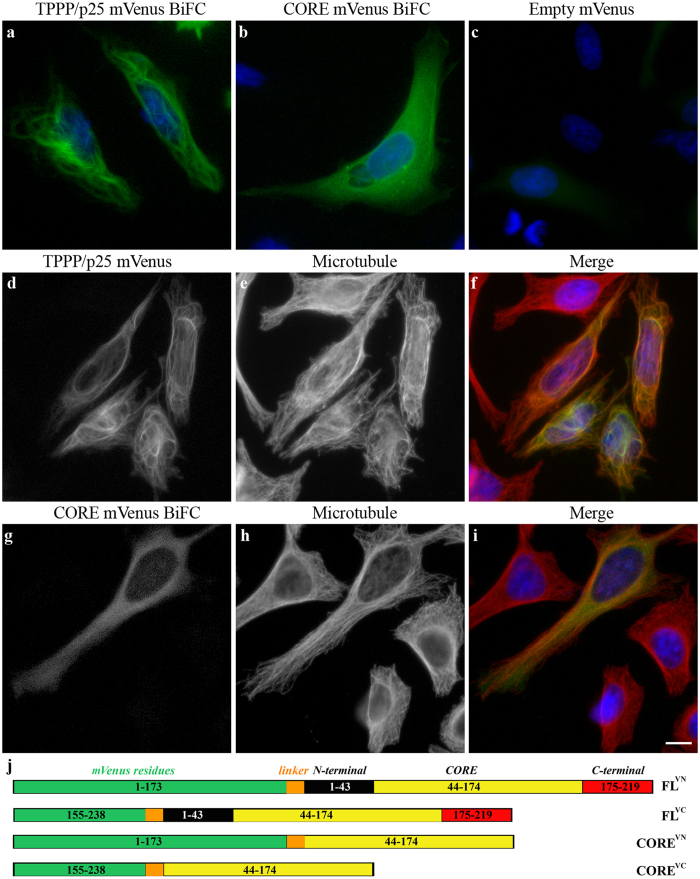
Intracellular dimerization of TPPP/p25 and CORE pairs in HeLa cells as detected by BiFC assay. BiFC signals of the N- and C-segments of the mVenus BiFC constructs coupled with FL (**a,d–f**) or CORE TPPP/p25 pairs (**b,g–i**) or without them (empty mVenus BiFC vectors) (**c**) as detected by immunofluorescence microscopy. Co-localization of the BiFC signals of TPPP/p25 (**f**) with the MT network and its lack in the case of CORE variant (**i**) is shown; the MT network is stained with Alexa546 (**e,h**). Note that the BiFC signal is aligned along the bundled MT network in TPPP/p25 expressing cells, however, the CORE segment-derived signal distributes homogeneously within the intracellular space of HeLa cells. Nuclei were counterstained with DAPI. Scale bar: 10 μm. BiFC signals were generated by co-transfection of the cells with the TPPP/p25 or CORE forms fused to mVenus BiFC constructs (a generous gift of Prof. Péter Várnai; Physiology Department, Semmelweis University, Budapest) according to the scheme (**j**).

We have performed a similar set of experiments using BiFC assay (mVenus 1–173 and 155–238 constructs) as described in the commented paper^1^ using mVenus vectors (cf. Fig. 3. legend) to test the associations of the FL-FL and CORE-CORE pairs at cellular level as well. As shown in Fig. 3a,b, the assembly of the mVenus pair (green fluorescence signal) is visible in both cases that correspond to the *in vitro* data (cf. Fig. 1). We have performed control experiments with the empty mVenus pair (Fig. 3c) in order to distinguish the BiFC signal produced by the TPPP/p25 forms from the non-specific BiFC signal generated by the intrinsic tendency of the two fluorescent protein domains to complement each other. In this control experiment low fluorescent signal could be detected as compared to that performed with the FL or CORE pairs. However, the intracellular localization of the BiFC signals was very distinct: in the case of FL protein it is aligned along the bundled MT network (Fig. 3d–f); while, the CORE segment-derived signal distributes predominantly homogeneously within the intracellular space of HeLa cells (Fig. 3g–i). These *in vivo* data suggest the involvement of the unstructured termini of TPPP/p25 in the cross-linking of the MT network. Periodical cross-linking of isolated MTs by recombinant TPPP/p25 were visualized by electron microscopy as well^11^. The electron microscopic images showed that the majority of MTs, 25–26 nm in diameter, formed bundles consisting of dozens of MTs decorated by rows of tiny projections and dense particles with a periodicity of about 16 nm. Similar electron microscopic analysis provided evidence for the periodic crosslinking of MTs by the tetrameric phosphofructokinase derived by the MT-bound dimeric enzymes^12^,^13^.

### Role of TPPP /p25 segments in the dimer-derived MT bundling

**Figure 4:**
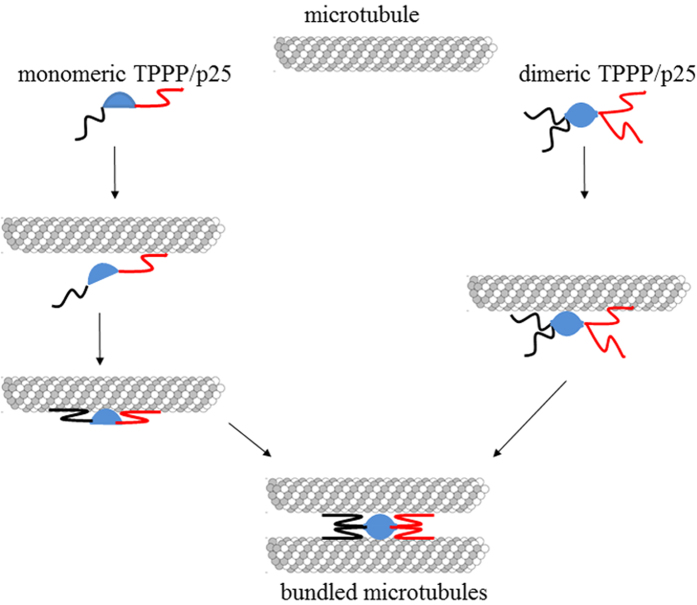
Tentative model for the development of the MT bundling in the course of dimerization of the TPPP/p25. All components (CORE and N- or C- termini) bind to MTs, but with different affinities based on Figs 1 and 2. Circular and semicircular forms represent dimeric and monomeric species, respectively; termini lines: back and red illustrate the N- and C-termini, respectively. The N- and C-termini could be positioned similarly or oppositely; however, the C-terminus binds with higher affinity as the N- one. The association of the C-terminal segment seems to be determining in the bundling process coupled without or with conformational changes.

Both the monomeric and dimeric TPPP/p25 bind to tubulin and MT inducing MT assembly, however, the polymerization potency of dimers seems to be higher; MT bundling activity is displayed by the dimers as illustrated in the tentative model. On the basis of our previous and present data, we have established a molecular model for the maturation of the bundled MT structure stabilized by TPPP/p25 dimers (Fig. 4).

## Discussion

TPPP/p25 has been recognized as a MAP that displays two crucial features: i) it does not have a 3D structure, namely it is disordered^2^,^6^,^14^; ii) it is enriched in inclusions of the neurons and oligodendrocytes in the cases of Parkinson’s disease and multiple system atrophy, respectively^15^; although in normal brain it is expressed primarily in oligodendrocytes^16^,^17^. The mechanism by which TPPP/p25 contributes to toxicity in Parkinson’s disease is related to its interaction with α-synuclein; their pathological complex has been considered as a potential drug target^5^,^15^. The physiological role of TPPP/p25 manifests itself in the differentiation of the progenitor oligodendrocytes as demonstrated by siRNA technique and specific microRNA (mir206)^17^. The differentiated oligodendrocytes are the major constituents of the myelin sheath.

The commented and critique papers, however, focus exclusively on the nature of the association of TPPP/p25 with the MT system. It has been well-documented earlier that TPPP/p25 promotes tubulin polymerization and the simultaneous bundling of the polymerized tubulin forms^2^ that stabilizes the intracellular MT ultrastructures such as perinuclear cage or aggresome^10^, and it makes them resistant against anti-microtubular agents such as nocodazole or vinblastine^7^,^10^. At cellular level bundling of the MT network by ectopically expressed TPPP/p25 in HeLa cells was demonstrated by both confocal and electron microscopies as well^10^, which revealed the existence of cross-linked MTs: the perinuclear cage consists of bundles of densely packed, parallel-aligned MTs running at different angles in close proximity to the nuclear membrane (cf. electron microscopic image).

Our experiments performed by immunofluorescent microscopy were reproduced by the Skoufias’ lab and presented in the DeBonis’ paper^1^ with references to our reported data. The single experiment that was not reproduced is the *in vitro* dimerization of the human recombinant TPPP/p25 in the absence of tubulin/MTs^1^. By studying carefully the differences of the experimental conditions used in the two labs we recognized a critical point causing the discrepancy, namely that all experiments performed in relation to the dimerization of TPPP/p25 by Skoufias and co-workers were carried out at reducing conditions (in the presence of DTT) that counteracted the stabilization of the dimers by disulphide bridges^4^. The spontaneous formation of the intermolecular covalent bridges at room temperature above 10 μM TPPP/p25 concentration can ensure the detection of dimers even by SDS/PAGE^4^. However, it is important to emphasize that TPPP/p25 dimers were detected in our lab, but not in Skoufias’s lab, by sandwich ELISA, isothermal titration calorimetry and size exclusion gel chromatography as well^4^,^8^.

In this paper we refer to a couple of data reported previously^4^,^5^,^8^; in addition, new sets of experiments have also been performed that allowed us to suggest a plausible mechanism for the role of the unstructured N- and C-termini of TPPP/p25 in the dimer-derived MT bundling. Our previous electron microscopic studies underlined that the MTs are cross-linked by TPPP/p25, formed bundles of 25–26 nm in diameter consisting of dozens of MTs decorated by rows of tiny projections and dense particles with a periodicity of about 16 nm were observed^2^,^11^. In fact, the particles, likely dimers, express significant stabilization effect, similar to one as detected in the case of tau protein^18^. The tau protein, a key MAP of the brain MT system occurs in soluble form in normal neuronal cells, and the soluble tau-tau interactions display stabilizing effect as well on the MT structures^19^,^20^. The formation of the pathological tau filaments has been reported, the development of which are promoted by the presence of MTs^19^. No TPPP/p25 filament formations have been detected as indicated in a recent paper as well to our knowledge; however, the formation of α-synuclein filaments has been established at substoichiometric TPPP/p25 concentration^21^.

In conclusion, our previous and the new data presented in this comment paper support the role of TPPP/p25 in the MT bundling in agreement with the title’s message of the Skoufias’s paper^1^ that focuses on the dimerization potency of this disordered, brain-specific MAP^2^,^4^. This issue does not seem to be affected by differences manifesting in the truncation sites: the Skoufias’s group chose shorter CORE segment based on bioinformatics analysis^1^ than we had published originally on the basis of multinuclear magnetic resonance study^6^. Based upon our previous and present *in vitro* and *in vivo* data obtained with the FL and truncated TPPP/p25 forms, we have established a molecular model for the maturation of the bundled MT structure stabilized by TPPP/p25 dimers (Fig. 4). We do not exclude other possible mechanisms such as the binding of the N- and C- termini to separate MTs. Nevertheless, the key issue is the crucial role of the unstructured termini, not in the dimerization but in the cross-linking of MTs, in which all segments (CORE, N- and C-termini) are involved with distinct binding affinity in a cooperative manner. The TPPP/p25-derived stabilization of the MT network seems to be of physiological importance in the development of projections in the course of the differentiation of oligodendrocytes^17^.

We are grateful for *Skoufias* and his co-workers for their work on the TPPP/p25 project, in fact, the paper in the *Scientific Reports*^1^ was a challenge which inspired us to reconsider our data and carry out further experiments in order to suggest a molecular mechanism regarding the MT bundling by TPPP/p25.

## Materials and Methods

### Antibodies

The following antibodies were used: mouse monoclonal tubulin antibody (Sigma T9026, clone DM1A), mouse monoclonal TPPP/p25 antibody^9^ and rat polyclonal TPPP/p25 antibody^15^.

### Tubulin preparation

Tubulin was prepared from bovine brain according to the method of Na and Timasheff^22^.

### Expression and purification of human recombinant wild type and mutant TPPP/p25 forms

Human recombinant TPPP/p25 forms and fragments possessing His-tags were expressed in E. coli BL21 (DE3) cells and isolated on HIS-Select™ Cartridge (Sigma-Aldrich) as described previously^5^,^15^. The purity of proteins was analysed by SDS-PAGE. Protein concentrations of the TPPP/p25 variants were determined on the basis of the absorbance at 280 nm using the extinction coefficients evaluated by ProtParam (http://web.expasy.org/protparam/), 10095 M^−1^ *cm^−1^ for FL TPPP/p25 and N-terminal truncated TPPP/p25; 5625 M^−1^ *cm^−1^ for C-terminal truncated TPPP/p25 and CORE, respectively.

### BiFC plasmids

The BiFC plasmids (pBiFC-VN1-173, pBiFC-VC155-238) were the gift of Prof. Péter Várnai (Semmelweis University, Budapest). To insert the FL TPPP/p25 cDNA in either the pBiFC-VN1-173 or pBiFC-VC155-238 the following primers for polymerase chain reaction (PCR) amplification were used: forward 5-GAAGGAGCTCGAGATATGGCTGACAAGGCTAAGC-3 and reverse 5-CCGTGGATCCCTACTTGCCCCCTTGCACCTTCTGGTCGTAGG-3 and pET21c-TPPP/p25 as a template. The PCR product, following purification and after digestion with XhoI-BamHI restriction enzymes, was inserted to XhoI and BamHI sites in the BiFC vectors. To insert the CORE segment of TPPP/p25 (TPPP/p25 delta 3–43/delta 175–219) cDNA in either the pBiFC-VN1-173 or pBiFC-VC155-238, the following primers for PCR amplification were used: forward 5- GGAATTC TATGGCTGCATCCCCTGAG-3 and reverse 5- ATGGATCCCTAGCCCGTGAACTTGGT-3′ and pET21c-TPPP/p25 as a template. The PCR product, following purification and after digestion with EcoRI-BamHI restriction enzymes, was inserted to EcoRI and BamHI sites in the BiFC vectors. The sequences of all construct were verified by restriction mapping and sequencing.

### ELISA

Sandwich ELISA assays were carried out similarly as described previously^8^. The plate was coated with 1 μg/mL (50 μL/well) mAb^9^ in 200 mM Na_2_CO_3_ buffer pH 9.6 overnight at 4 °C. The wells were blocked with 1 mg/mL bovine serum albumin in phosphate-buffered saline (PBS) for 1 h at room temperature. Next, the plate was incubated with serial dilutions of 5 μM TPPP/p25 for 1 h at room temperature in PBS. Where indicated, TPPP/p25 was pre-incubated with 100 μM DTE for 30 min at room temperature. In another experimental setup, different TPPP forms at 1 μM concentration were added to the plate coated with mAb in the absence and presence of DTE. Alternatively, 1 μM FL TPPP/p25 or CORE TPPP/p25 was preincubated with 100 μM DTE for 30 min at room temperature, then added to the plate coated with mAb; after removing the excess DTE, the different TPPP/p25 forms were added, and dimers were detected by biotinylated mAb. Then the plate was sequentially incubated with biotinylated mAb (1 μg/mL) and peroxidase conjugated avidin (Calbiochem) (2.5 μg/mL). Both antibodies were in PBS buffer containing 1 mg/mL bovine serum albumin, and incubated for 1 h at room temperature. Between each incubation step the wells were washed thrice with PBS containing 0.05% Tween 20 for 10 min. The presence of antibodies was detected using o-phenylenediamine as substrate. The reaction was stopped after 10 min with 1 M H_2_SO_4_, and the absorbance was read at 490 nm with an EnSpire Multimode Reader (Perkin Elmer). The binding constants (K_d_) were evaluated from the saturation curves by non-linear curve fitting assuming single binding site hyperbola model using the Origin 8.0 software.

### Turbidity measurements

The assembly of tubulin (7 μM) was assessed in polymerization buffer (50 mM 2-(N-morpholino) ethanesulfonic acid buffer pH 6.6 containing 100 mM KCl, 1 mM DTE, 1 mM MgCl_2_ and 1 mM ethylene glycol tetraacetic acid) at 37 °C. The tubulin polymerization into MTs was induced by the addition of 3 μM of the different TPPP/p25 forms. In another set of experiments, the polymerization of 10 μM tubulin into MTs was induced by the addition of 20 μM paclitaxel then subsequently 3 μM TPPP/p25 proteins were added to the solution. The optical density was monitored at 350 nm by a Cary 100 spectrophotometer (Varian).

### Pelleting experiments

Tubulin (5 μM) was incubated with the different TPPP/p25 forms (10 μM) for 10 min at 37 °C in polymerization buffer. In another set of experiments, 10 μM paclitaxel-stabilized MTs was incubated with the different TPPP/p25 forms (10 μM) for 10 min at 37 °C in polymerization buffer. After centrifugation at 17000 g for 15 min at 37 °C, the pellet and the supernatant fractions were separated. The pellet fraction was washed and resuspended in buffer. Then the fractions containing 2-mercaptoethanol were analysed by SDS-PAGE, separated on a 13.5% gel and stained with Coomassie Brilliant Blue R-250.

### Cell culture, transfection and manipulation

HeLa cells (ATCC^®^ CCL-2™, American Type Culture Collection) were grown in Dulbecco’s modified eagle medium supplemented with 10% fetal calf serum and 100 μg/ml kanamycin in a humidified incubator at 37 °C with 5% CO_2_. Cells were grown on 12-mm-diameter glass coverslips for microscopic analysis. For the evaluation of the BiFC signal, HeLa cells were transfected with different mVenus constructs of TPPP/p25 (0.3 μg of each plasmid) using Turbofect (Invitrogen) transfection reagent according to the manufacture’s protocol. Monoclonal tubulin antibody (Sigma T9026) and an Alexa 546 conjugated mouse secondary antibody (Thermo Fisher Scientific A11030) were used for the detection of the tubulin signal. Nuclei were counterstained with 4′,6-diamidino-2-phenylindole (DAPI). Images of the mounted samples were acquired on a Leica DM IL 500 microscope equipped with Leica DFC 395 FX camera and HBO 100w lamp. The equipment software was Leica Application Suite 4.4.0. Chroma UV filter set (No. C40888), Chroma 41028 HQ NB GFP filter set (No. C21116) and Leica filter N2.1 (No. 513832) was used for DAPI, BiFC and Alexa 546 signal acquisition, respectively, using a HCX FL Fluotar 40x/0.75 (dry) objective.

### Statistics

Each data point represents the mean ± standard deviation (SD) from three independent experiments.

## Additional Information

**How to cite this article**: Oláh, J. *et al*. Further evidence for microtubule-independent dimerization of TPPP/p25. *Sci. Rep.* **7**, 40594; doi: 10.1038/srep40594 (2017).

**Publisher's note:** Springer Nature remains neutral with regard to jurisdictional claims in published maps and institutional affiliations.
